# CpG-ODN and MPLA Prevent Mortality in a Murine Model of Post-Hemorrhage-*Staphyloccocus aureus* Pneumonia

**DOI:** 10.1371/journal.pone.0013228

**Published:** 2010-10-07

**Authors:** Antoine Roquilly, Laetitia Gautreau, Jean Pierre Segain, Pierre de Coppet, Véronique Sebille, Cédric Jacqueline, Jocelyne Caillon, Gilles Potel, Corinne Lejus, Régis Josien, Karim Asehnoune

**Affiliations:** 1 Laboratoire UPRES EA 3826 «Thérapeutiques cliniques et expérimentales des infections», Faculté de médecine, Université de Nantes, Nantes, France; 2 Centre Hospitalier Universitaire de Nantes, Service anesthésie réanimation chirurgicale, Hôtel Dieu-HME, Nantes, France; 3 Unité Mixte de Recherche 643, Institut National de la Santé et de la Recherche Médicale, Nantes, France; 4 Unité Mixte de Recherche 1280 “Physiologie des Adaptations Nutritionnelles”, Institut National de Recherche Agronomique, Université de Nantes, Nantes, France; 5 Cellule de Biostatistique – Cellule de promotion à la recherche clinique & EA 4275, Université de Nantes, Faculté de Pharmacie, Nantes, France; 6 Centre Hospitalier Universitaire de Nantes, Laboratoire d'Immunologie, Nantes, France; 7 Institut de Transplantation –Urologie – Néphrologie (ITUN), Nantes, France; Institut de Pharmacologie et de Biologie Structurale, France

## Abstract

Infections are the most frequent cause of complications in trauma patients. Post-traumatic immune suppression (IS) exposes patients to pneumonia (PN). The main pathogen involved in PN is Methicillin Susceptible *Staphylococcus aureus* (MSSA). Dendritic cells () may be centrally involved in the IS. We assessed the consequences of hemorrhage on pneumonia outcomes and investigated its consequences on DCs functions. A murine model of hemorrhagic shock with a subsequent MSSA pneumonia was used. Hemorrhage decreased the survival rate of infected mice, increased systemic dissemination of sepsis and worsened inflammatory lung lesions. The mRNA expression of Tumor Necrosis Factor-alpha (TNF-α), Interferon-beta (IFN-β) and Interleukin (IL)-12p40 were mitigated for hemorrhaged-mice. The effects of hemorrhage on subsequent PN were apparent on the pDCs phenotype (reduced MHC class II, CD80, and CD86 molecule membrane expression). In addition, hemorrhage dramatically decreased CD8^+^ cDCs- and CD8^-^ cDCs-induced allogeneic T-cell proliferation during PN compared with mice that did not undergo hemorrhage. In conclusion, hemorrhage increased morbidity and mortality associated with PN; induced severe phenotypic disturbances of the pDCs subset and functional alterations of the cDCs subset. After hemorrhage, a preventive treatment with CpG-ODN or Monophosphoryl Lipid A increased transcriptional activity in DCs (TNF-α, IFN-β and IL-12p40) and decreased mortality of post-hemorrhage MSSA pneumonia.

## Introduction

In developed countries, severe trauma remains the leading cause of death, particularly among individuals younger than 30 years old [1], [2]. Despite the development of new antibiotics and significant advances in rescue and intensive care medicine, infections are the most frequent cause of complications and death in severely injured patients [3], [4]. The average cost of these infections in intensive care units remains very high despite the use of prevention strategies [5]. Among infections, pneumonia (PN) is a major cause of morbimortality [6], [7]. We [8] and others [9] have reported that methicillin-susceptible *Staphylococcus aureus* (MSSA) is the main pathogen involved in post-traumatic PN. A marked depression of cell-mediated immune function, known as post-traumatic immune suppression (IS), plays a role in sepsis after severe trauma [10].

The major features of post-trauma IS include 1) decreased *ex vivo* production of lipopolysaccharide (LPS)-induced proinflammatory cytokines [11], [12] and 2) decreased human leucocyte antigen (HLA)-DR expression (antigen presentation capacity) on antigen-presenting cells (APCs) [13]. Major surgery, multiple injuries, and severe sepsis lead to decreased monocyte HLA-DR expression [13]–[15]. Decreased monocyte HLA-DR expression is the only IS marker that correlates with infection and clinical outcomes in severe trauma patients [14], [16].

Dendritic cells (DCs) are the most potent antigen-presenting cells and are endowed with the unique capacity to activate naïve T cells [17]. DCs are thus central in the initiation of adaptive immunity. They are also able to detect pathogen-associated molecular patterns (PAMPs) through large numbers of pattern recognition receptors (PRRs) including Toll-like receptors (TLRs). Stimulation of immature DCs by several TLR agonists (via TLR4 and TLR9) triggers DCs maturation. Several subsets of DCs have been described in the mouse spleen: a main population called conventional DCs (cDCs) that can be separated into CD8^+^ and CD8^−^ subsets, and a population of plasmacytoid DCs (pDCs). The pDCs are specialized in the production of type I interferon (IFN), whereas cDCs produce large amounts of interleukin (IL)-12.

The goals of the present study were 1) to determine the consequences of hemorrhage on subsequent MSSA PN, 2) to investigate the effect of hemorrhage on splenic DCs functions, and 3) to evaluate the ability of TLR agonists to reverse mortality of post-hemorrhage pneumonia. Our results demonstrate that hemorrhage decreased survival of mice challenged with MSSA PN, increased systemic dissemination of the infection, and worsened lung damage associated with PN. Hemorrhaged mice developed severe phenotypic disturbances of the pDCs subset and functional alterations of the cDCs subset. Interestingly, CpG-ODN and MPLA increased the transcription of cytokines in DCs and prevented mortality associated with post-hemorrhage PN.

## Results

### Pilot study

**Figure 1:**
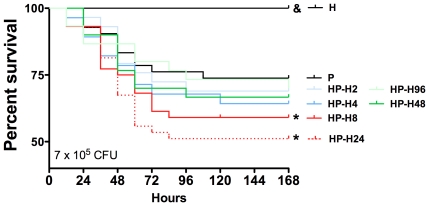
Effects of hemorrhage on timing-based mortality. Three groups of mice were studied: H group (hemorrhaged animals); P group (methicillin-susceptible *S. aureus* (MSSA)-induced pneumonia only; n = 15) and HP group (animals hemorrhaged before MSSA-induced pneumonia; n = 15). Pneumonia was induced in mice with MSSA (7×10^5^ CFU) 2, 4, 8, 24, 48, or 96 hours after hemorrhage (HP-H2, -H4, -H8, -H24, -H48, and -H96 groups, respectively) and survival was compared with mice with P group and H group. Survival rates are expressed as percentage and are representative of three independent experiments. & P<0.05 *versus* all others; *P<0.05 *versus* P group.

To determine the effects of hemorrhage on survival in mice, PN was induced with MSSA (7×10^4^, 7×10^5^, or 7×10^6^ colony forming units [CFUs]) 24 hours after hemorrhagic shock (HP group) and compared with mice in which PN was induced without hemorrhagic shock (P group). As shown in Figure S2A–C, survival was decreased when PN was preceded by hemorrhagic shock with the lowest inoculum level (7×10^4^ CFU; 100% *versus* 89% for P and HP groups, respectively; P<0.05) and with the intermediate inoculum (7×10^5^ CFU; 72% *versus* 51%, P<0.05), whereas all animals died before hour 60 with the highest inoculum tested (7×10^6^ CFU). Post-hemorrhagic susceptibility to sepsis is a dynamic process; therefore, hemorrhage-induced mortality was evaluated over time (Figure 1). PN was induced 2, 4, 8, 24, 48, or 96 hours after hemorrhagic shock (HP-H2, -H4, -H8, -H24, -H48 and -H96 groups, respectively). Survival was not significantly lower in mice that underwent hemorrhagic shock 2 or 4 hours before PN induction (group P, 73%; HP-H2, 69%; HP-H4, 64%; P>0.05 *versus* P group), whereas survival was significantly decreased in mice that underwent hemorrhagic shock 8 or 24 hours before PN (HP-H8, 58%; HP-H24, 51%; P<0.05 *versus* P group). However, longer intervals between hemorrhagic shock and PN induction did not decrease survival (HP-H48, 67%; H-H96, 71%) (Figure 1).

### Main study

Based on the results of the pilot study, PN was induced with 7×10^5^ CFU MSSA (P group) 24 hours after hemorrhage (HP group) (Figure S1).

#### Hemorrhagic shock increased weight loss and other biological consequences of PN

**Figure 2:**
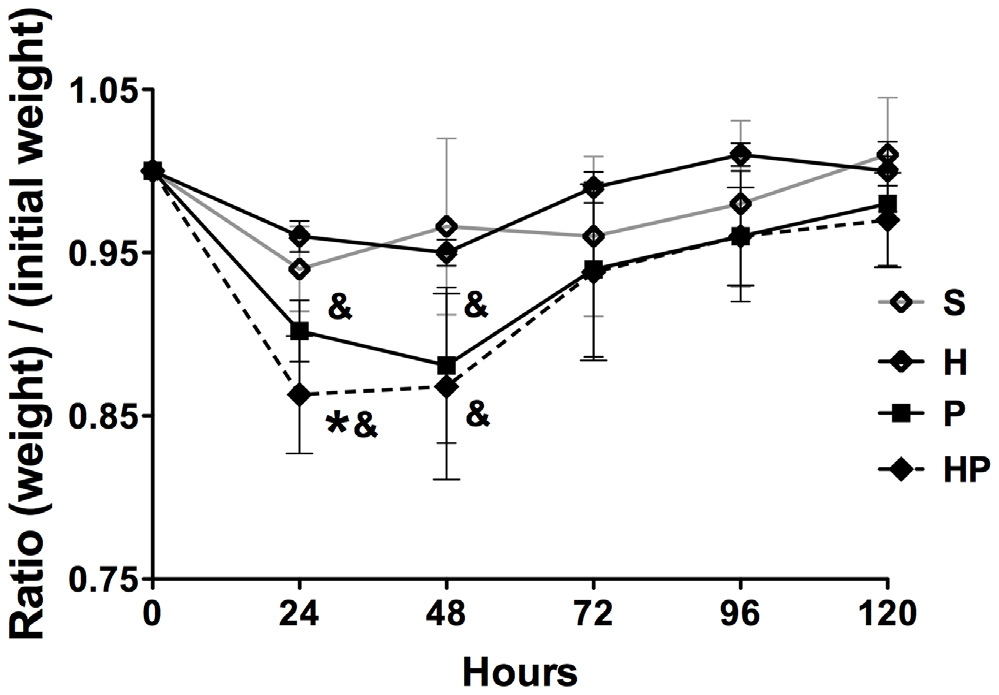
Hemorrhagic shock increases weight loss after methicillin-susceptible *S. aureus* (MSSA) pneumonia. Mice received sham treatment (S; cardiac puncture without blood collection), Hemorrhage (H), MSSA–induced pneumonia alone (P), or hemorrhage before MSSA–induced pneumonia (HP), and were weighed daily for 5 days. Data are representative of three independent experiments (each group, n = 6). Data are expressed as mean ± SEM. & P<0.05 versus S and H groups, *P<0.05 versus P group.

The effects of hemorrhage on PN outcomes were assessed. Mice in the HP group lost more weight in the first 24 hours (Figure 2) and experienced more severe metabolic acidosis and smaller drop in blood glucose (Table 1) compared with mice in group P. Weight and blood gas appeared unchanged in H group compared with group S except for bicarbonate and base excess levels (Table 1).

**Table 1:** Hemorrhagic shock worsens biological consequences of methicillin-susceptible *S. aureus* (MSSA) pneumonia.

	Sham (S) group	Hemorrhage (H) group	Pneumonia (P) group	Hemorrhage-Pneumonia (HP) group
Lactate (mmol/l)	3.5±0.5	4.2±1.3	4.6±1.3&	6.0±1.5**^&^** *
pH	7.34±0.03	7.32±0.04	7.26±0.03&	7.22±0.01**^&^** *
HCO_3_- (mmol/l)	28.5±1.1	24.5±2.1&	26.8±1.6	24.5±3.0**^&^** *
BE (mmol/l)	+0.4±0.2	−1.4±0.2&	−0.9±0.8	−2.8±0.7**^&^** *
PcO_2_ (kPa)	6.4±1.1	5.8±1.6	3.4±1.2&	3.8±1.3&
PcCO_2_ (kPa)	7.3±0.9	6.8±0.4	8.5±0.7	9.1±1.3
SvO_2_ (%)	59±12	41±12&	29±18&	35±19&
Blood glucose (mmol/l)	1.87±0.29	1.88±0.45	0.81±0.3&	1.36±0.23**^&^** *

*BE: Base Excess, PcO2: Central venous Pression in O2, PcCO2: Central venous Pression in CO2, SvO2: Venous saturation in oxygen.* Blood samples were collected via cardiac route (right atria) 24 hours after hemorrhage (H group) or MSSA pneumonia onset (P and HP groups).

**^&^** *P≤0.05 versus S group,*

*P≤0.05 versus P group.

#### Hemorrhagic shock aggravated lung lesions and slowed recovery after PN

**Figure 3:**
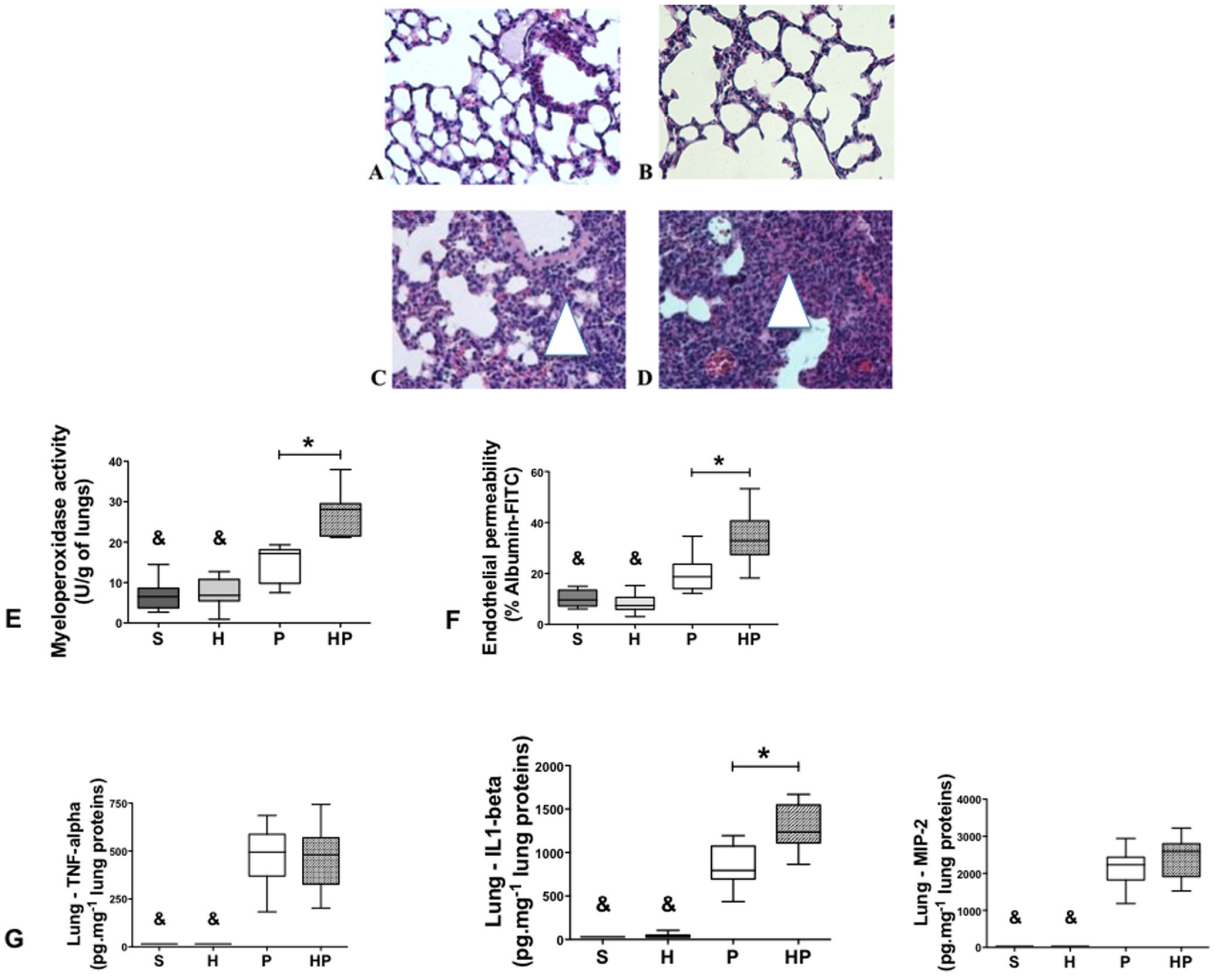
Hemorrhagic shock prior to methicillin-susceptible *S. aureus* (MSSA) pneumonia worsens lung damage. Lungs were harvested 24 hours after intratracheal instillation (S, P and HP groups) or hemorrhagic shock (H group). (***A, B, C, D***) Hematoxylin and eosin-stained sections of lungs (n = 3; magnification ×20) from mice that underwent the (***A***) sham procedure (S group), (***B***) Hemorrhage (**H**)**,** (***C***) MSSA-induced pneumonia (P group), or (***D***) hemorrhage before MSSA-induced pneumonia (HP group). Light microscopy revealed increased immune cell infiltration (purple stained) in group HP (***D***) compared with S, H and P groups. (***E***) Neutrophil accumulation assessed by myeloperoxidase activity in lung homogenates. (***F***) Vascular permeability was assessed in whole lung by measuring albumin-FITC passage through lung capillary. (***G***) Concentrations of TNF-α, IL-1β, and MIP-2 were assessed in lung homogenates. Data are representative of three independent experiments (each group, n = 6). Boxes represent median (interquartile range). & P<0.05 *versus* P and HP groups; *P<0.05 *versus* P group.

Because hemorrhagic shock worsens PN outcomes, we assessed the lungs histology. In both sham-treated group (S group) and H group, lung tissue was characterized by thin-walled air spaces with a single pneumocyte layer (Figure 3A, B). In contrast, immune cell infiltrates (macrophages and neutrophils) were detected and alveolar layers were thicker in the P group 24 hours after MSSA injection (Figure 3C). Histological recovery began at day 4 with increasing aeration of the lung; lungs were almost normal at day 7 (Figure S3C, E, G). Histological lesions appeared sooner (as soon as 12 hours vs. 24 hours) and for a longer duration (7 days vs. 4 days) in the HP group compared with the P group (Figure 3D and Figure S3D, F, H).

Twenty-four hours after MSSA injection, neutrophil accumulation, as assessed by myeloperoxidase activity (Figure 3D), and endothelial lesions, as assessed by endothelial permeability to albumin-FITC (Figure 3E), were increased in the HP group compared with the P group. Lung concentrations of tumor necrosis factor (TNF)-α, IL-1β, and macrophage inflammatory protein (MIP)-2 were all elevated in the P and HP groups compared with the S and H groups (Figure 3F). In addition, lung production of IL-1β was higher in the HP group compared with P group (Figure 3F).

#### Hemorrhagic shock increased systemic dissemination of MSSA

**Figure 4:**
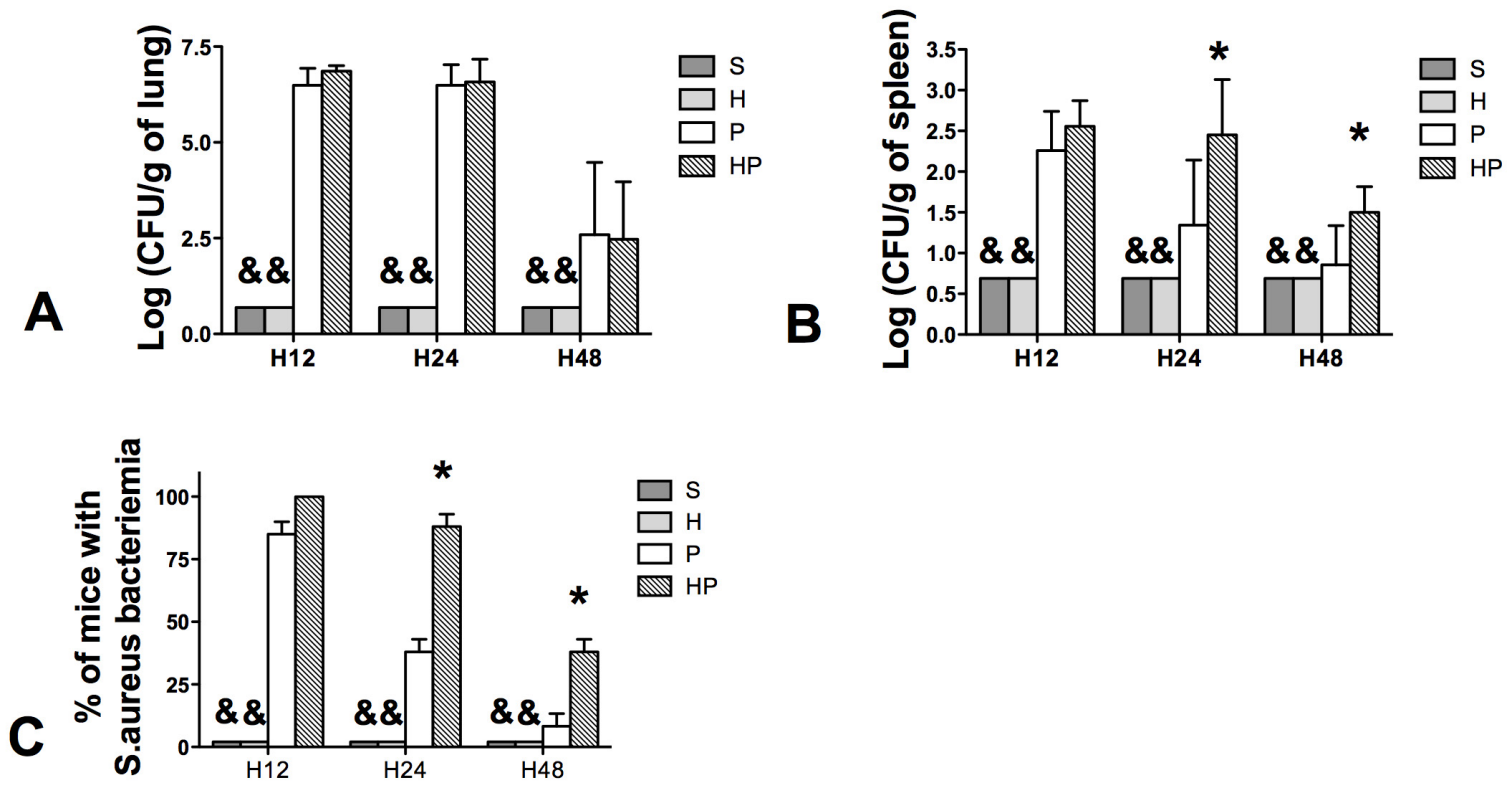
Hemorrhagic shock prior to methicillin-susceptible *S. aureus* (MSSA) pneumonia induces a bacteriemia. Four groups of mice were studied: sham-treated (S group), Hemorrhaged mice (H group), MSSA-induced pneumonia (P group), and hemorrhage before MSSA-induced pneumonia (HP group). Mice were sacrificed 12, 24, or 48 hours after pneumonia onset. MSSA counts in (***A***) lungs and (***B, C***) spleen homogenates were performed after culture on specific media. Detection threshold was 0.7 colony forming unit (CFU) per gram of tissue. MSSA counts were always below the threshold in the S and H groups. Data are representative of three independent experiments (each group, n = 6). Data are presented as mean ± SEM (**A, B**) or percentage ± SEM (**C**). & P<0.05 *versus* P and HP groups; *P<0.05 versus group P.

Because PN lung lesions were increased after hemorrhagic shock, local and systemic bacterial burden were evaluated 12, 24, and 48 hours after PN. MSSA was not detected in the S and H groups; however, lung bacterial load were not different in groups HP and P (Figure 4A). Spleen cultures showed that systemic dissemination of infection (MSSA CFUs) was higher at 24 and 48 hours after MSSA injection in group HP compared with group P (Figure 4B). At each time point, the percentage of mice exhibiting systemic dissemination was higher in group HP than in group P (Figure 4C).

#### Hemorrhagic shock aggravated blood hyporeactivity to LPS observed in PN

**Figure 5:**
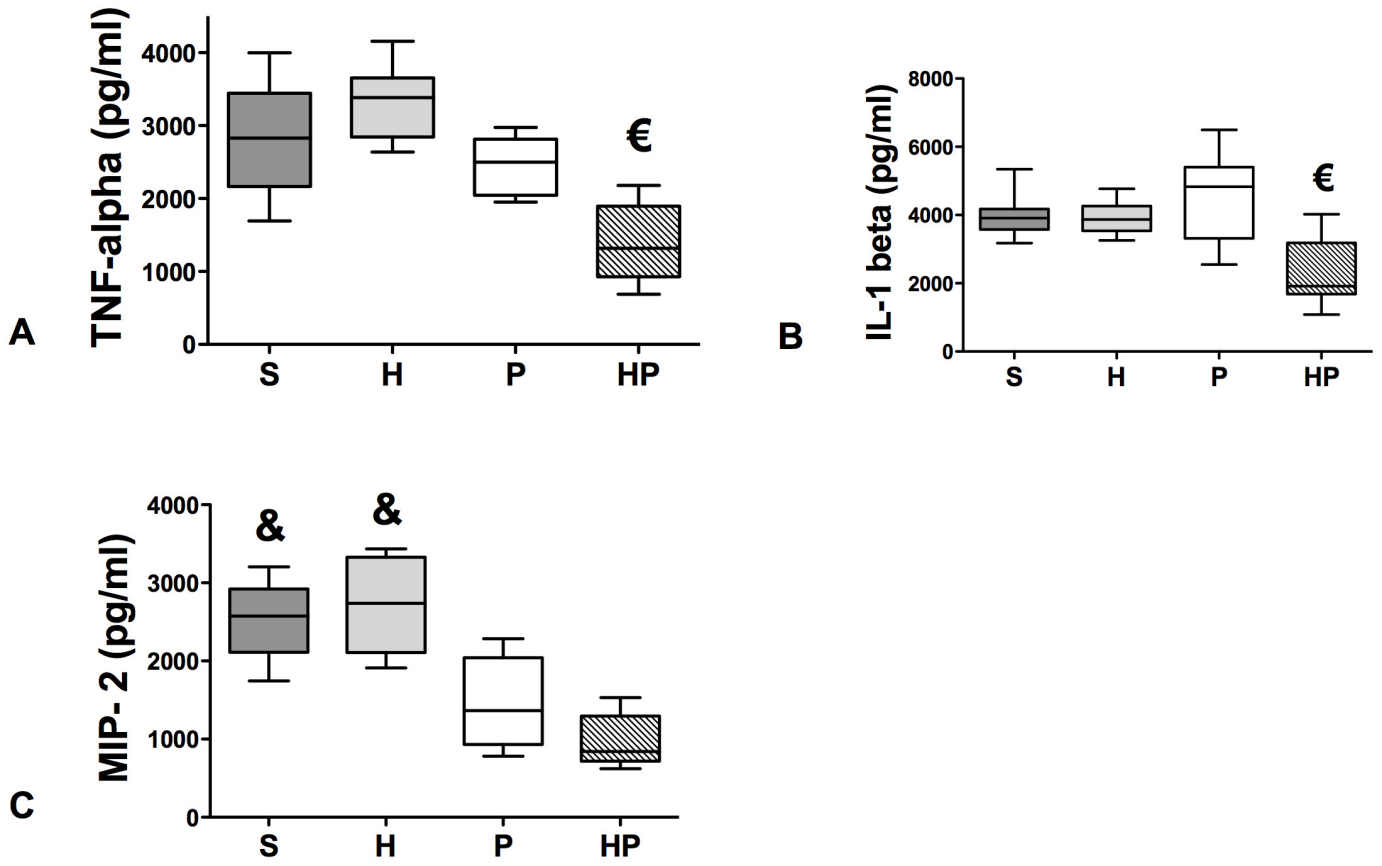
Whole Blood cells cultures. Hemorrhagic shock prior to methicillin-susceptible *S. aureus* (MSSA) pneumonia worsens peripherical blood reactivity after ex vivo LPS stimulation. Four groups of mice were studied: sham-treated (S group), hemorrhaged mice (H group); MSSA-induced pneumonia (P group), and hemorrhage before MSSA-induced pneumonia (HP group). Whole blood was exposed to LPS from *E.coli O111 B4* for 24 hours and the following cytokines were assessed in the cell culture medium: (*A*) TNF-α, (*B*) IL-1β, and (*C*) MIP-2. Cytokine concentrations in the absence of LPS stimulation were always below the detection threshold (30 pg/ml). Data are representative of three independent experiments (each group, n = 6). Boxes represent median (interquartile range). € P<0.05 *versus* all other groups; & P<0.05 *versus* P and HP groups.

As previously described [10], we measured cytokine production in whole blood cells cultures stimulated by LPS. TNF-α and IL-1β production was significantly lower in the HP group compared with all other groups (Figure 5A, B), whereas MIP-2 production was decreased in both HP and P groups compared with groups S and H (Figure 5C).

DCs link innate immunity to adaptive immunity and may be critically involved in hemorrhage-induced PN mortality. We therefore sought to determine whether hemorrhagic shock preceding PN could affect spleen DCs numbers, phenotypes, and function.

#### Hemorrhagic shock downregulates inflammatory cytokine mRNA expression in total spleen DCs

**Figure 6:**
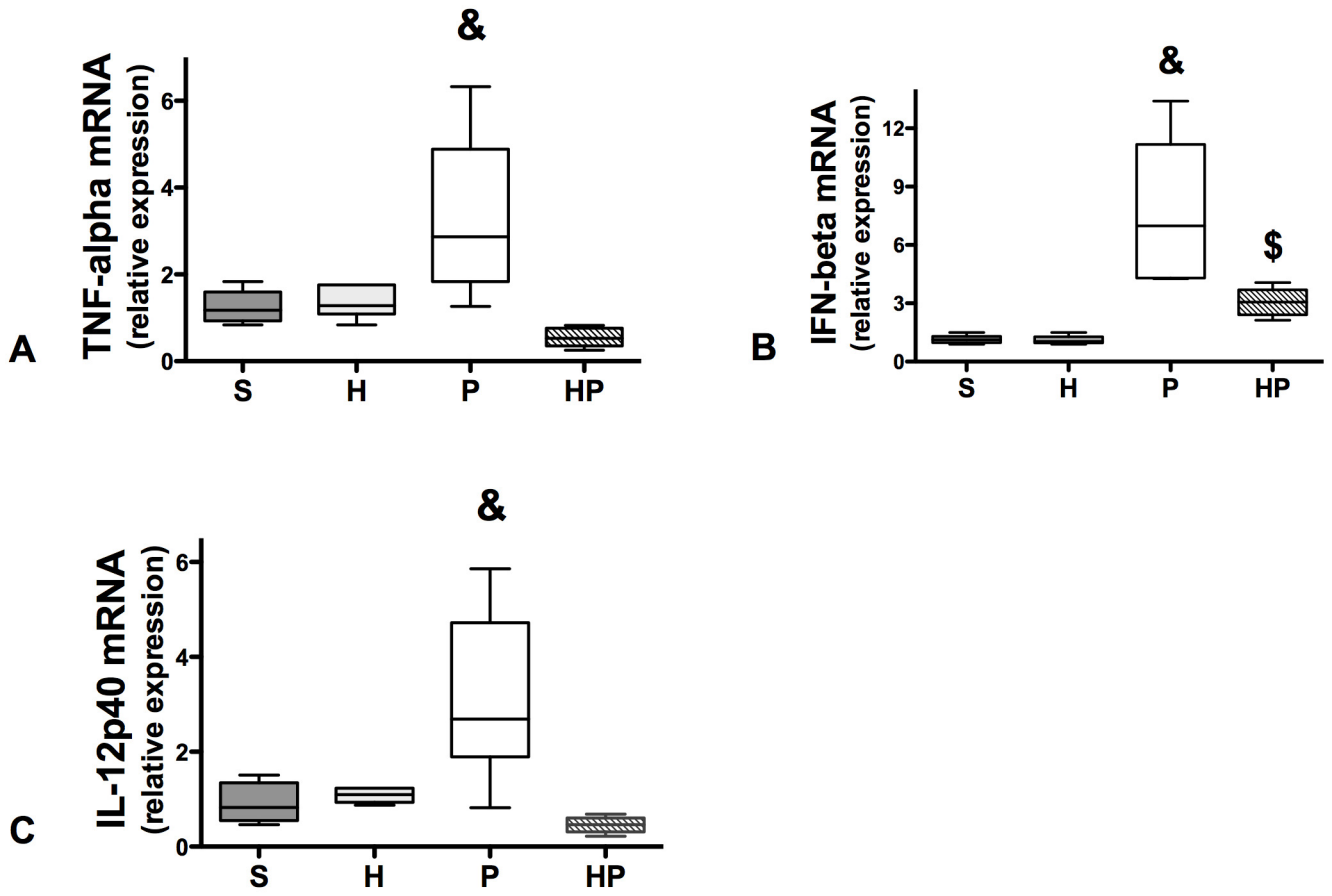
Hemorrhagic shock prior to methicillin-susceptible *S. aureus* (MSSA) pneumonia markedly decreases cytokine mRNA levels in total dendritic cells (DCs). Four groups of mice were studied: sham-treated (S group), hemorrhaged mice (H group), MSSA-induced pneumonia (P group), and hemorrhage before MSSA-induced pneumonia (HP group). Mice were sacrificed 6 hours after MSSA injection. Real-time RT-PCR analysis of (*A*) TNF-α, (*B*) IFN-β, (*C*) IL-12p40 was performed. In each group, mRNA was extracted from CD11c cells positively selected from cell suspension obtained from enzymatic spleen digestion. Data are representative of three independent experiments (each group, n = 6). Boxes represent median (interquartile range). & P<0.05 *versus* all others, $ P<0.05 *versus* S and H groups.

We assessed time-dependent mRNA expression of inflammatory cytokines by real-time RT-PCR in total spleen DCs. Cytokine mRNA levels peaked 6 hours after MSSA injection (see Figure S4). TNF-α, IFN-β, and IL-12 mRNA levels were significantly increased in group P compared with group S (Figure 6A–C and Figure S4), indicating that DCs are involved in the innate immune response to MSSA PN. Mice that underwent hemorrhagic shock prior to MSSA-induced PN (group HP) demonstrated markedly decreased TNF-α, IFN-β, and IL-12p40 mRNA expression compared with P group (Figure 6A–C). Hemorrhage alone did not affect mRNA expression compared with S group.

#### Hemorrhagic shock performed before PN altered the phenotype of pDCs but not cDCs

**Figure 7:**
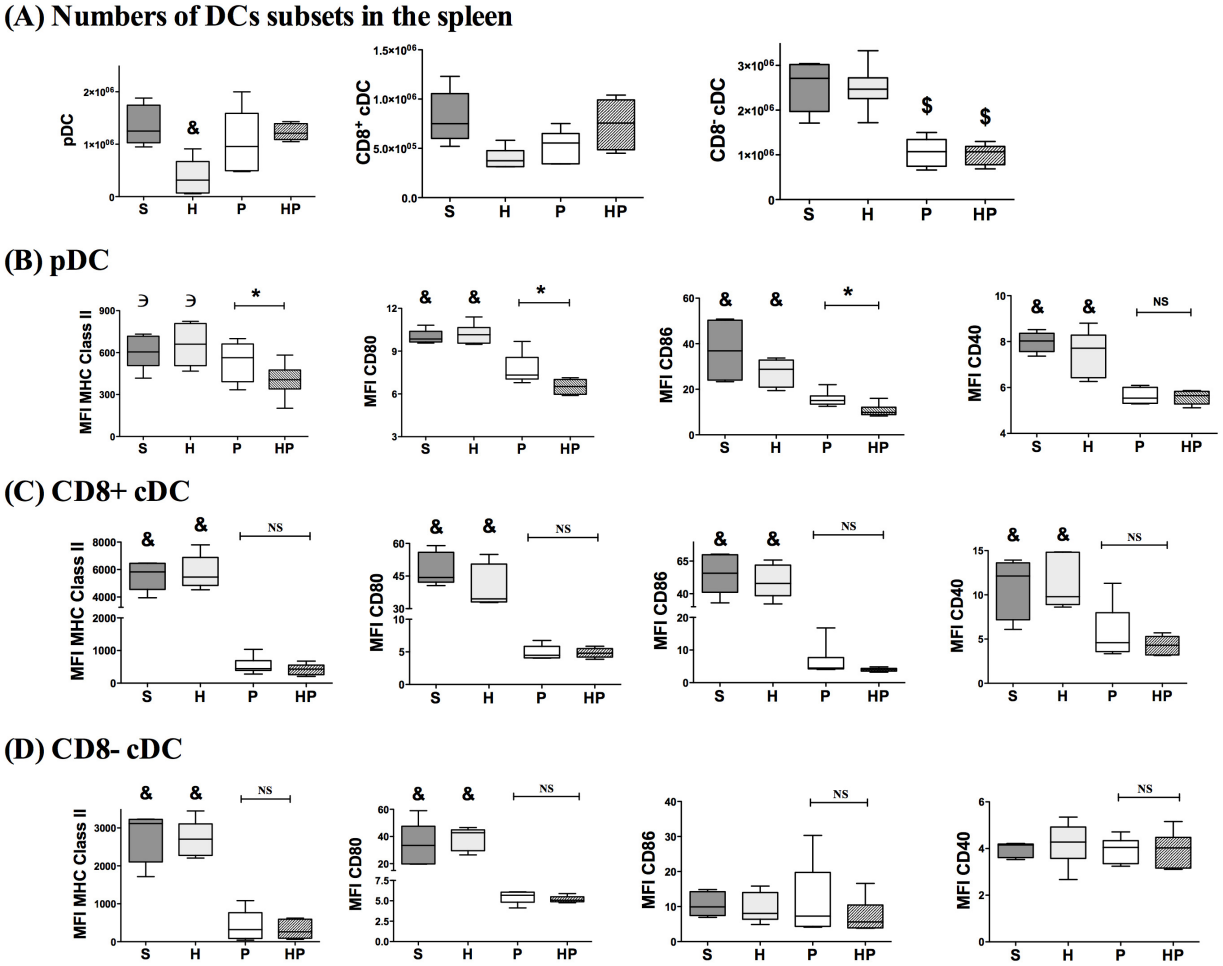
Hemorrhagic shock prior to methicillin-susceptible *S. aureus* (MSSA) pneumonia induced phenotypic alterations of plasmacytoid dendritic cells (pDCs). Four groups of mice were studied: sham-treated (S group), hemorrhaged mice (H group), MSSA-induced pneumonia only (P group), and hemorrhage before MSSA-induced pneumonia (HP group). Spleens were harvested 24 hours after sepsis onset. (***A***) Cells obtained from spleen homogenates were counted. The percentage of each subset of DCs was determined by a FACS analysis and number of each DCs subset was calculated. DCs subsets were defined by specific membrane markers: B220 and siglec H for pDCs, CD11c and CD8 to differentiate CD8^+^ conventional DCs (cDCs) and CD8^−^ cDCs. Mean fluorescence intensity (MFI) of MHC class II, CD80, CD86, and CD40 molecules on (***B***) pDCs, (***C***) CD8^+^ cDCs, and (***D***) CD8^−^ cDCs was assessed. Data are representative of two independent experiments (each group, n = 6). Boxes represent median (interquartile range). & P<0.05 *versus* P and HP groups, € P<0.05 *versus* HP group, *P<0.05.

To further evaluate potential alterations of DCs, we determined the numbers and phenotypes of individual spleen DCs subsets. The number of CD8^−^ cDCs was decreased in PN-infected groups, whereas number of pDCs was decreased in H group (Figure 7A). The pDCs from HP mice exhibited a significant decrease in MHC class II, CD80, and CD86 molecule levels compared with the S, H and P groups (Figure 7B), whereas CD40 expression was decreased in both HP and P groups compared with the S and H groups. Regarding CD8^+^ cDCs, MHC-class II and costimulatory molecules were downregulated in the HP and P groups compared with the S and H groups (Figure 7C). Finally, MHC class II and CD80 molecules, but not CD86, were downregulated similarly in CD8- cDCs of groups HP and P compared with S and H groups (Figure 7D). Therefore, hemorrhage did not significantly affect the numbers of DCs subsets following PN infection; however, hemorrhage induced phenotypic alterations in pDCs, but not cDCs.

#### Hemorrhagic shock before PN decreased the ability of cDCs to induce T-cell proliferation

**Figure 8:**
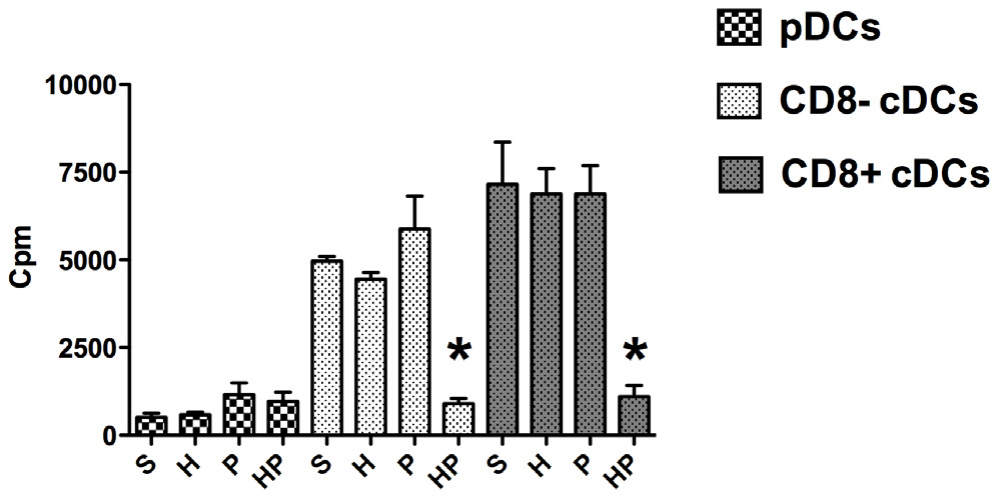
Mixed lymphocyte reaction assay. Four groups of mice were studied: sham-treated (S group), hemorrhaged mice (H group), methicillin-susceptible *S. aureus* (MSSA)-induced pneumonia (P group), and hemorrhage before MSSA-induced pneumonia (HP group). DCs subsets were defined by specific membrane markers: B220 and siglec H for plasmacytoid DCs (pDCs), CD11c and CD8 to differentiate CD8^+^ conventional DCs (cDCs) from CD8^−^ cDCs. DCs subsets were sorted and treated for 24 hours with CpG 1826 (5 µM). Each DCs subset was cultured with allogeneic CD4^+^ and CD8^+^ T cells (ratio DCs:T cells, 1∶25) for 3 days. For each DCs subset, determining thymidine incorporation within 8 hours assessed T-cell proliferation. Data are representative of three independent experiments (each group, n = 6). Histograms represent mean ± SEM. *P<0.05 *versus* all others.

To further characterize potential functional abnormalities of DCs after hemorrhage, the abilities of each DCs subset (pDCs, CD8^+^ cDCs, and CD8^−^ cDCs) to induce T-cell proliferation were tested using an allogeneic mixed lymphocyte reaction (MLR) assay. As previously described [18], DCs subsets were separated by fluorescence-activated cell sorting (FACS) 24 hours after MSSA injection and stimulated overnight with TLR9 ligand to induce maturation before the MLR assay. T-cell proliferation induced by CD8^+^ and CD8^−^ cDCs did not differ between the S, H and P groups. In contrast, the ability of both cDCs subsets to induce T-cell proliferation was dramatically impaired in the HP group (Figure 8). Taken together, these data indicate that hemorrhagic shock potently decreased the antigen-presenting function of mature cDCs. As previously reported [19], pDCs are relatively poor stimulators of T cells and no significant differences in pDCs were observed among the four treatment groups.

#### CpG-ODN 1668 and MPLA restored transcriptional activity in DCs and reversed excess PN mortality caused by hemorrhagic shock

**Figure 9:**
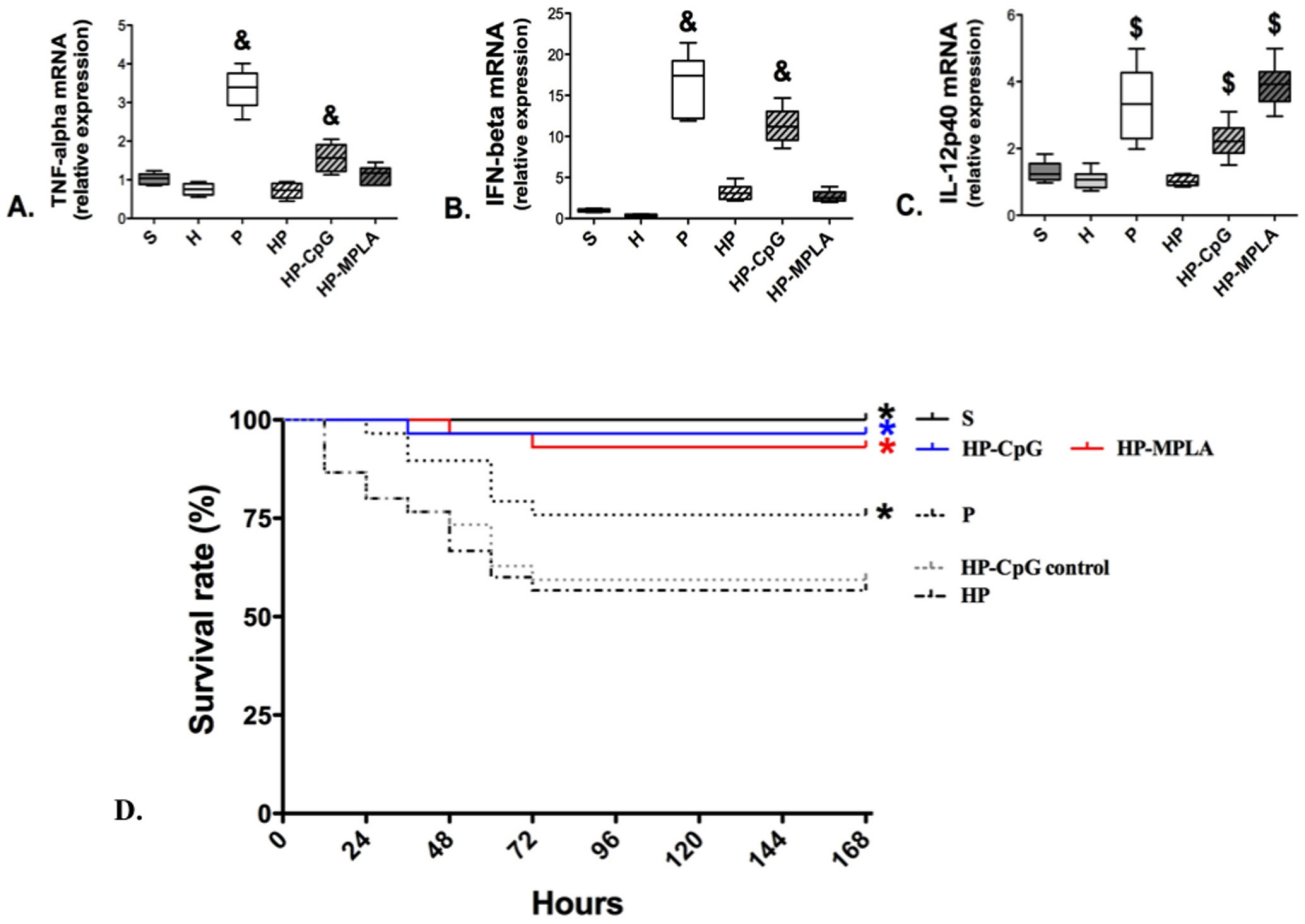
Effect of CpG-ODN or MPLA on cytokine mRNA levels in dendritic cells (DCs). Six groups of mice were studied: sham-treated (S group), hemorrhagic shock (H group), methicillin-susceptible *S. aureus* (MSSA)-induced pneumonia (P group), hemorrhage before MSSA-induced pneumonia (HP group), injections of CpG-ODN 1668 or of MPLA were performed immediately after resuscitation of hemorrhage: HP-CpG and MPLA groups respectively. (***A, B, C***) Mice were sacrificed 6 hours after MSSA injection. Real-time RT-PCR analysis of (***A***) TNF-α, (***B***) IFN-β, (***C***) IL-12p40 was performed. In each group, mRNA was extracted from CD11c cells positively selected from cell suspension obtained from enzymatic spleen digestion. Boxes represent median (interquartile range). & P<0.05 *versus* S, H, HP and HP-MPLA groups; $ P<0.05 *versus* S, H and HP groups. (***D***) The survival rate in each group was monitored twice a day for 7 days (168 hours). Survival rates are presented as percentages. * P<0.01 *versus* HP group. Data are representative of three independent experiments (each group, n = 5 to 6 mice for A, B and C; n = 8 mice for D).

Because hemorrhage affected DCs subsets, the ability of TLR9 agonist CpG-ODN 1668 (64 µg/mouse; group HP-CpG) and TLR4 agonist monophosphoryl lipid A (MPLA, 50 µg/mouse; group HP-MPLA) to prevent the increased post-hemorrhage PN mortality was evaluated. To mimic a clinical scenario, TLR agonists were intravenously injected immediately after the resuscitation of hemorrhage and before PN induction. The mRNA levels of TNF-α, IFN-β and IL-12p40 were increased in DCs from HP-CpG group compared with HP group (Figure 9A, B, C). The mRNA levels of IL-12p40 were increased in HP-MPLA group compared with HP group (Figure 9C). As shown in Figure 9D, the survival rate was significantly increased in mice that received MPLA (93% in HP-MPLA vs. 60% in group HP; P<0.01) or CpG-ODN (97% in HP-CpG; P<0.01 compared with 60% in group HP). Injection of the control CpG-ODN (64 µg/mouse, group HP-CpG control) did not improve survival compared with the HP group.

## Discussion

Our results indicate that (i) hemorrhage increases PN-induced morbidity and mortality, (ii) hemorrhage induces pDCs phenotypic alterations, as well as important defects in cDCs-induced T-cell proliferation (iii) CpG-ODN (TLR9 agonists) and MPLA (TLR-4 agonists) prevent hemorrhage-induced PN mortality, and restore the transcriptional activity of DCs.

PN remains the most frequent cause of complications and death in patients with severe hemorrhage trauma [18], [19], and MSSA is the primary pathogen involved in post-traumatic PN [20]. We chose a model that mimics the clinical scenario of a severe trauma patient presenting with a hemorrhagic shock and subsequent MSSA PN.

Hemorrhagic shock profoundly suppresses many immune functions, and numerous clinical studies indicate that a marked depression of cell-mediated immune functions persists for 1 or 2 weeks after the initial hemorrhagic insult [10], [12], [16]. This post-hemorrhagic susceptibility to sepsis has been associated with two major immune dysfunctions: first, a decreased leukocyte capacity to produce proinflammatory cytokines in response to LPS *ex vivo* [10], [12], [16]; second, a decreased capacity of antigen-presenting cells (APCs) to present antigens [13]. Several studies indicate that post-traumatic immune dysfunction plays a role in the development of subsequent infections [13], [14], [16]. Animal models have shown that hemorrhage performed before intraperitoneal infection increased mortality up to 100% [21], [22]. In a murine model, hemorrhage produces an immunosuppressed state characterized by an increased susceptibility to *Pseudomonas aeruginosa* PN [23]. Our findings confirm that hemorrhagic shock before MSSA PN increases mortality, probably through systemic dissemination of the pulmonary MSSA infection.

Neutrophils have been shown to play a central role in the innate immune response to hemorrhage because they are primed for increased lung sequestration and cytotoxic activity [24]. Consistent with these data, our results demonstrate that hemorrhage increases pulmonary neutrophil infiltration induced by MSSA PN. Other surrogate markers of lung damage, such as lung proinflammatory cytokines (mainly IL-1β) and vascular permeability, were also exacerbated by hemorrhage in the present study. Despite this apparently overwhelming inflammatory response, as in another murine model of sepsis-induced immune dysfunction [25], bacterial clearance in the lungs was not affected by hemorrhage whereas systemic bacterial dissemination was constant.

Studies [10], [12], [26] have demonstrated that blood leukocytes from trauma or septic patients produced lower levels of proinflammatory cytokines in whole blood cultures stimulated ex vivo with LPS compared with controls. In the present study, hemorrhagic shock performed before MSSA PN also resulted in a marked decrease of blood reactivity to LPS. The association of an overwhelming inflammation into the lungs with a blood hyporeactivity to LPS in *ex vivo* whole blood cell cultures has been described in human trauma patients [27] (compartmentalization of the inflammatory response between lung and systemic circulation). Moreover, lung production of cytokines depends on several cellular types (neutrophils, epithelial cells, macrophages) [24] whereas such a production in whole blood cultures depends mainly on monocytes [28].

Antigen presentation to T cells requires both signal I (MHC class II-peptide complex) and signal II (co-stimulatory molecules such as CD80 and CD86), which are both provided by professional APCs [29]. Interestingly, an early decrease in HLA-DR and CD86 membrane expression on circulating monocytes predict subsequent infections after trauma [16]. DCs are the most potent APCs and have the unique ability to activate naïve T cells [30]. In a murine model of hemorrhage, Kawasaki et al. [31] reported that decreased expression of MHC class II and CD83 molecules on splenic DCs following hemorrhage was associated with a defect in DCs-induced T-cell proliferation. To the best of our knowledge, the effects of hemorrhage followed by a subsequent infection have not been reported to date.

Several DCs subsets have been described in rodents and humans [17], [32]. PDCs are known to be important in the antiviral response. These cells secrete large amounts of type I IFN upon viral stimulation, exhibit a restricted TLR repertoire specialized in nucleic acid recognition (TLR7 and TLR9) and have a weak capacity for antigen presentation [33], [34]. In mice, cDCs express a large panel of TLRs including TLR9, and are critically involved in bacterial control. Two subsets of cDCs have been described: CD8+ cDCs produce considerable amounts of IL-12, induce strong T helper cell (Th)1 responses, and efficiently cross-present antigens to CD8+ T cells; CD8- cDCs produce IL-10 and drive the Th2 response. The innate immune response relies on close collaboration among the three DCs subsets [17]. Alterations of total DCs after hemorrhage have been reported, whereas specific subset alterations remain poorly studied. A decreased number of cDCs was reported in trauma patients [35], as well as in patients with severe sepsis [15]. Accordingly, in a model of peritonitis, both cDCs subsets were markedly decreased in the spleens of infected mice [36]. In the present study, the effects of hemorrhage on subsequent PN (HP group) were apparent on the pDCs phenotype (reduced MHC class II, CD80, and CD86 molecule membrane expression) with no significant cDCs phenotypic alterations. In addition, our findings demonstrate that post-hemorrhage IS dramatically decreased CD8^+^ cDCs- and CD8^−^ cDCs-induced allogeneic T-cell proliferation during PN compared with mice that did not undergo hemorrhage. These results could be explained either by the reported decreased cytokines mRNA production or by a resistance to TLR-induced maturation in cDCs during post hemorrhage pneumonia. We would like to point out here that the phenotype of DCs was assessed directly ex vivo on spleen cells whereas MLR were performed after stimulating DCs in vitro with TLR ligands to induce maturation [37]. Therefore, these experiments cannot be clearly correlated.

It is important to note that although human cDCs do not express TLR9, mouse pDCs and cDCs express and respond to TLR9. Our data suggest that hemorrhagic shock induces *ex vivo* unresponsiveness to TLR9 agonists in cDCs by an unknown mechanism, which was apparent during the subsequent sepsis. Lack of response to TLR4 ligands was observed as the decreased production of inflammatory cytokines in whole blood cells cultures stimulated with LPS *ex vivo*. This is consistent with studies showing impaired NF-κB activation in monocytes from trauma patients in response to LPS [10]. Of particular interest, leukocyte deactivation described in trauma-patients was not a generalized phenomenon but depended on the stimulus and the signaling pathway under study [10]. The relatively poor ability of pDCs to stimulate allogeneic T cells in the MLR assay was expected; pDCs have been consistently shown to exhibit lower antigen-presenting capacity compared with cDCs in vitro [33]. In fact, the role of pDCs in stimulating naïve T cells remains controversial [38]. However, pDCs can be a major source of inflammatory cytokines such as type I IFN and TNF-α, and have also been shown to cooperate with cDCs to induce the adaptive immune response. Given the phenotypic alterations observed in pDCs, and the decreased mRNA levels of IFN-β (type I IFN) in DCs of the HP group it is likely that pDCs functions were impaired. Additional studies are needed to characterize the functional status of pDCs after hemorrhage.

The results presented here suggest that *in vivo* functional alterations of all DCs subsets could be a major therapeutic target for prevention of post-hemorrhage sepsis. We hypothesized that systemic stimulation of TLRs during resuscitation could circumvent post hemorrhage immunological dysfunction, thereby improving survival after pneumonia. CpG-ODN is a TLR9 agonist with potent immunostimulatory effects on B lymphocytes and DCs [39]. In a murine model of sepsis, prophylactic injection of CpG-ODN decreased mortality through an enhanced innate effector cell response [40]. A type B CpG-ODN (CpG-ODN 1668) was tested in an attempt to stimulate DCs before sepsis onset, and to decrease the mortality of post hemorrhage MSSA PN. It has been demonstrated that activation of TLR9 signaling pathway could be deleterious in a murine model of sepsis [41]. However, these results were obtained in a polymicrobial model of peritonitis by using TLR9-/- animals. Our approach was different because we administered TLR-9 agonists (CpG-ODN) before infection, in an attempt to prevent hemorrhage-induced MSSA PN, and CpG-ODN treatment decreased the mortality as well as restoring the transcriptional activity of DCs. TLR4 is expressed primarily on cDCs (CD8^+^ and CD8^−^) and not expressed on pDCs in mice and humans [42]. Because cDCs exhibit functional defects following hemorrhage, the TLR4 agonist MPLA may also be a good candidate for treating post hemorrhage susceptibility to sepsis. Indeed, MPLA is known to enhance antigen presentation by cDCs via selective activation of the intracellular Toll/Interleukin-1 receptor-domain-containing adapter-inducing interferon (TRIF) pathway, without inducing an overwhelming cytokine secretion [43], [44].

Consistent with our hypothesis, prophylactic injections of MPLA or CpG-ODN increased the transcription of cytokines in DCs and increased the survival rate in a murine model of post-hemorrhage PN, suggesting that trauma-induced sepsis in humans may potentially be prevented by MPLA or CpG-ODN. These immunostimulatory molecules are already approved for human use as vaccine adjuvants and appear to safely and efficiently induce DCs maturation [45].

In conclusion, hemorrhagic shock decreases survival to MSSA PN and induces alterations of DCs subsets. Further studies are needed to better characterize the mechanism by which CpG-ODN and MPLA may alter the DCs functions during post hemorrhage-pneumonia.

## Materials and Methods

### Ethics Statement

All experiments were conducted in accordance with the Principles of Laboratory Animal Care (NIH publication No 86-23, revised 1985) and French regulations addressing animal experiments. The committee of animal ethics of the University of Nantes approved all animal experiments in this study.

### Animals

Male BALB/cJ (20–24 g) were purchased from Janvier Laboratories, Laval, France. Mice were maintained on a 12-hour light/dark cycle with free access to food and water.

### Hemorrhage procedure

The hemorrhage/resuscitation protocol for this model was previously described [46], [47]. Male BALB/cJ were anesthetized with 1.5 ml isoflurane (Baxter, Maurepas, France). Transthoracic cardiac puncture was performed with a 29-gauge needle to withdraw 30% of the calculated blood volume (0.3 ml/10 g body weight) over 45 seconds. The shed blood volume (SBV) was restored by a retro-orbital plexus injection 60 min later. Arterial pressure returned to baseline with restitution of the SBV [47].

### Preparation of bacterial inocula

MSSA strains (ATCC 29213) were grown for 18 hours in tryptic soy broth at 37°C. Immediately before use, cultures were washed twice (centrifuged at 1000×*g* for 10 min at 37°C) and diluted in sterile isotonic saline to be calibrated by nephelometry. Bacterial concentration (CFU) was controlled by quantitative culture.

### PN procedure

PN was induced according to a previously published method [48]. Mice were anesthetized with isoflurane and placed in dorsal recumbency. Transtracheal insertion of a 24-gauge feeding needle was used to inject 70 µl of the bacterial preparation. The rate of intratracheal inoculation reaches 100% with this procedure.

### Clinical monitoring

Whole blood glucose was measured using a hand-held glucometer. Blood gas was determined using central venous blood harvested through the right heart chambers.

### Assessment of bacterial growth and dissemination

Lungs and spleen were mechanically homogenized under sterile conditions. Organ homogenates were subjected serial 10-fold dilution and cultured at 37°C on Chapman medium to avoid growth of non-staphylococci bacteria. After a 48-hour incubation, colonies were counted and results expressed as log_10_ CFU per gram of organ. Bacterial colonies were identified by specific tests.

### Histological analysis

Both lungs were removed and immediately placed in 4% formalin. Formalin-fixed tissues were processed, stained with hematoxylin and eosin, and then analyzed by microscopy.

### Myeloperoxidase assay

The myeloperoxidase assay was performed as previously described [49]. Lungs were harvested, frozen in liquid nitrogen, and stored at −80°C. Lungs were mechanically homogenized on ice for 25 seconds in 1 ml of potassium phosphate (50 mM) with N-ethylmaleimide (10 mM). The homogenate was washed twice (centrifuged at 12,000×*g* for 30 min at 4°C), suspended in 1 ml of potassium phosphate buffer (50 mM) containing 0.5% of hexadecyl trimethylamonium, and sonicated on ice water for 180 seconds. Heat shock was performed for 2 hours at 60°C, and then samples were centrifuged at 12,000×*g* for 10 min. The H_2_O_2_-dependant oxidation of o-dianisidine was determined by measuring absorbance at 460 nm. Supernatant myeloperoxidase activity was normalized to lung weight.

### Lung endothelial permeability

The lung endothelial permeability assay was performed as previously described [48]. Mice were given a 2-mg intraperitoneal injection of fluorescein isothiocyanate (FITC)-conjugated albumin (Sigma, Lyon, France). After 2 hours, the lungs were harvested, mechanically homogenized in 1 ml of isotonic saline, and then centrifuged at 4000×*g* for 10 min. Blood was collected via right ventricular puncture and centrifuged at 4,000×*g* for 10 min. FITC-albumin was measured in 100-µl supernatant aliquots obtained from lung homogenates and blood by fluorometry at 480 nm. Lung endothelial permeability was calculated according to the validated equation: 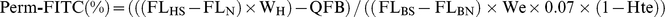 (see Table S1).

### Preparation of lung homogenate for enzyme-linked immunosorbent assay

Enzyme-linked immunosorbent assay (ELISA) analysis of lung homogenates was performed as previously described [49]. Immediately after removal, weighed lung samples were mechanically homogenized in cold lysis buffer (1× phosphate buffered saline [PBS, pH 7.4], 0.1% Triton X-100) containing 1 mM protease inhibitor cocktail (Sigma). Homogenates were centrifuged at 12,000×*g* for 20 min at 4°C. Supernatant was then collected and stored at −80°C until analysis. Protein concentration in each sample was determined using the BCA™ protein assay kit, according to manufacturer's instructions (Pierce, Rockford, IL. United States).

### Cell cultures for LPS reactivity assessment

LPS reactivity of cell cultures was assessed as previously described [46], [50]. Monocyte cytokine secretion was induced in whole blood culture by LPS. Briefly, blood samples were diluted 1∶5 in RPMI-1640 medium (Laboratoire de Biotechnologies, Reims, France) supplemented with 100 U ml^−1^ penicillin (Panpharma, Fougères, France) and 100 µg ml^−1^ streptomycin (Sigma). The diluted blood was cultured in 24-well plates (500 µl per well) with or without LPS (*E. coli* O111:B4, 10 µg/ml; Sigma) in a 5% CO_2_ incubator for 24 hours at 37°C. Supernatants were collected by centrifugation at 12,000×*g* for 2 min and stored at −80°C before cytokine determination by ELISA.

### Determination of cytokine levels in samples

TNF-α, IL-1β, and MIP-2 concentrations were quantified with ELISA kits according to manufacturer's instructions (R&D Systems, Lille, France).

### Spleen cell suspension

Spleens were minced and digested in 2 mg/ml collagenase D (Roche Diagnostics, Meylan, France) in RPMI 1640 supplemented with 1% fetal calf serum (FCS) for 25 min at 37°C. EDTA (10 mM) was added for the last 5 min of digestion. The cell suspension was then filtered through a 80-µm filter and washed in PBS (centrifuged at 12,000×*g* for 10 min at 4°C).

### Real-Time Reverse Transcription Polymerase Chain Reaction

Real-time reverse transcription polymerase chain reaction (RT-PCR) was performed as previously described [51]. Spleen cells were incubated with anti-mouse CD11c-coated magnetic beads for 15 min at 4°C. After washing, CD11c+ cells were purified by positive selection using magnetic affinity cell sorting (MACS) separation columns (Miltenyi Biotec, Paris, France). This procedure routinely yielded cell populations with purity up to 90%. Total RNA was isolated from purified spleen CD11c+ cells with TRIzol reagent (Invitrogen, Cergy Pontoise, France) and treated for 45 min at 37°C with 2 U of RQ1 DNase (Promega, Lyon, France). RNA (1 µg) was reverse-transcribed with Superscript III Reverse Transcriptase (Invitrogen). The cDNA (1 µl) was subjected to real-time RT-PCR in a Bio-Rad iCycler iQ system using the QuantiTect SYBR Green PCR kit (Qiagen, Courtaboeuf, France). Thermal cycling conditions consisted of 45 cycles of 30 seconds at 95°C followed by 30 seconds at 60°C. Mice primer sequences for TNFα, IFN-β, IL-12, IL-10 and glyceraldehyde-3-phosphate dehydrogenase (GAPDH) were designed using “Primer-BLAST” primer design software on the National Center for Biotechnology Information (NCBI) website (see Table S2 for primer sequences). GAPDH was used to normalize gene expression. Relative gene expression was calculated by the 2^−ΔΔ^ Ct method [52] using samples from the sham group as calibrator samples.

### Antibody, flow cytometry, and cell sorting

Flow cytometry and cell sorting were performed as previously described [37] (see Figure S5 for example of gating). Monoclonal antibodies (mAb) used for cytometry and/or cell sorting were obtained from BD Biosciences (United States, Franklin Lakes, NJ, United States): anti-CD3 (1452C11), anti-CD8α (53.6-7), anti-CD11c (HL3), anti-CD19 (1D3), anti-CD40 (3123), anti-CD45R (B220, RA3-6B2), anti-CD80 (16-10A1), anti-CD86 (GL1), anti-IAd (class II MHC, AMS-32.1), anti-NK11 (PK136), anti-Ter 119 (Ter119), anti-T-cell receptor (TCR)β (H57.597). The anti-siglec H was obtained from eBiosciences (San Diego, CA. United States). All mAbs were conjugated to FITC, phycoerythrin (PE), PECy7, peridinin-chlorophyll-protein-complex (PerCP)-Cy5.5, allophycocyanin (APC), or biotin (detected with APC-Cy-labeled streptavidin) (BD Biosciences). Flow cytometry was performed on a FACS LSR II (BD Biosciences) and cell sorting was performed on a FACS Aria (BD Biosciences).

For phenotypic analysis, spleen cells were labeled with antibodies against CD8, CD11c, B220, Siglec H, and lineage (Lin) antigens (CD3, CD19, TCRβ, NK1.1, Ter119). DCs activation was assessed by the following biotinylated mAbs: CD80, CD86, CD40 or MHC-class II (IAd). A total of 4×10^6^ cells were analyzed for each biotinylated Ab. Dead cells were excluded by 4',6-diamidino-2-phenylindole (DAPI) staining. Data were analyzed using FlowJo software (Treestar, United States) (see Figure S5 for an example).

For DCs cell sorting, after collagenase digestion and Ficoll gradient centrifugation, spleen cells were first incubated with unconjugated CD3 (17A2, BD Biosciences) and CD19 (1D3, BD Biosciences) rat mAbs, which are specific for T and B cells, respectively. Positive cells were magnetically depleted with sheep anti-rat IgG-conjugated beads (Dynabeads, Invitrogen), and sheep/goat α-mouse IgG conjugated beads. The remaining cells were labeled with antibodies against B220, CD19, CD11c, and CD8α. This procedure routinely yielded populations with levels of purity up to 98%.

For the MLR assay, T cells were recovered from the lymph nodes of C57BL/6 mice. Cell suspensions were depleted of erythrocytes, myeloid, natural killer, CD8^+^, and B cells with the Pan T-cell Isolation kit (Miltenyi Biotech, Paris, France) according to manufacturer's instructions. Sorted DCs subsets were stimulated with CpG-ODN 1826 (5 µM) for 24 hours and then cultured with allogeneic T cells (DCs:T-cell ratio of 1∶25) in round-bottom 96-well plates with RPMI 1640 (10% Fetal Calf Serum, 100 mg/mL streptomycin, 100 IU/mL penicillin). Cells were cultured for 3 days at 37°C in 5% CO_2_, and then pulsed for the last 8 hours with 0.5 µCi of [^3^H]thymidine (GE Healthcare) per well. The cells were then harvested onto glass fiber filters and [^3^H]thymidine incorporation was measured using standard scintillation procedures (Packard Institute).

### Toll Like Receptor Agonists

CpG-ODN 1668, CpG-ODN 1168 control and MPLA were purchased from Invivogen (Toulouse, France).

### Statistical analysis

SAS 9.1 (Evry-Gregy sur Yerres, France) and GraphPad prism (La jolla, CA. Uinited States) software were used for statistical analysis. For the pilot study, potential experimental effects were tested using Cox proportional-hazards regression models by introducing the corresponding covariate into the model. In case of a non-significant effect, the corresponding covariate was not included in subsequent analyses. Survival rates were compared using the log-rank test or exact log-rank test as appropriate. Normally distributed data were expressed as mean ± standard error of the mean (SEM) and analyzed using analysis of variance (ANOVA) and Student's *t*-test. Continuous non-parametric variables were expressed as median (interquartile range) and were compared using the Kruskal Wallis test for multiple comparisons. In case of significance, the Mann-Whitney test was used for inter-group comparison. P<0.05 was considered to be statistically significant.

## Supporting Information

Figure S1 Diagrammatic representation of the six experimental groups in the main study. In the sham group (S), cardiac puncture was performed without blood collection or resuscitation. Volume-controlled hemorrhage was performed by cardiac puncture (0.3 ml/10 g body weight) and resuscitation with shed blood was performed after 60 min (groups Hemorrhage [H] and Hemorrhage-Pneumonia [HP]). After 24 hours, mice underwent intratracheal instillation of 7×10^5^ CFU (70 µl) of methicillin-susceptible *S. aureus* (groups Pneumonia [P] and HP) or sterile PBS (group S). Intravenous infusion of CpG-ODN (64 µg/mouse, HP-CpG group) or MPLA (50 µg/mouse, HP-MPLA group) were performed immediately after resuscitation. Twenty-four hours* after intratracheal instillation, mice were euthanized and specimens were collected. *unless otherwise stated.
(1.02 MB PDF)

Figure S2 Effects of hemorrhage on inoculum-based mortality. Two groups of mice were studied: HP group (animals hemorrhaged before methicillin-susceptible *S. aureus* (MSSA)-induced pneumonia; n = 15) and P group (MSSA-induced pneumonia only; n = 15). Survival rates are expressed as percentage and are representative of three independent experiments. Twenty-four hours after hemorrhage for HP group, pneumonia was induced with (A) 7×10^4^ CFU, (B) 7×10^5^ CFU, or (C) 7×10^6^ CFU of MSSA. Survival was monitored twice a day for 7 days. *P<0.05 versus P group.
(3.00 MB TIF)

Figure S3 Evolution of histological findings following sepsis onset. Four groups of mice were studied (each group, n = 3): naive, sham-treated (S), methicillin-susceptible *S. aureus* (MSSA)-induced pneumonia only (P), and hemorrhage before MSSA-induced pneumonia (HP). Formalin-fixed tissues were processed, stained with hematoxylin and eosin, and analyzed by microscopy (magnification,×20). Representative lung histology for (A) normal lung (naive), (B) lung at 24 hours post sterile instillation (S group). The parenchyma is shown along with a series of images obtained 12, 96, and 168 hours after pneumonia onset in (C, E, G) for group P, respectively, and (D, F, H) for group HP. Aggregates of purple-stained immune cells were observed as early as 12 hours postinfection (arrow) and were more numerous in group HP compared with group P at all time points. These data established a murine model of MSSA pneumonia that closely mimics the clinical and histological findings for human patients.
(3.00 MB TIF)

Figure S4 Time-dependent cytokine mRNA expression in spleen dendritic cells (DC) following sepsis onset. Mice in which pneumonia was induced by methicillin-susceptible *S. aureus* (P group). Mice were sacrificed 1, 6, or 12 hours after Meticillin Susceptible Staphylococcus aureus injection. Then mRNA was extracted from CD11c+ cells positively selected in spleen cells suspension. Data are representative of two independent experiments (n = 8). Boxes represent median (interquartile range). *P<0.05.
(3.00 MB TIF)

Figure S5 Phenotypic characterization of mouse spleen dendritic cell (DC) subsets. Spleen cells from sham-treated mice (S group), methicillin-susceptible *S. aureus* (MSSA)-infected mice (P group), hemorrhage-shocked and MSSA-infected mice (HP group) were labelled with antibodies against lineage antigens (CD3e, CD19, TCRb, Ter119, NK1.1) after DAPI staining. Conventional dendritic cell (cDC) and plasmacytoid cell (pDC) subsets were identified within the lineage-negative population as CD11chigh, CD8^+^, or CD8^−^ cells and B220- siglec H+ cells respectively. Expression of CD80, CD86, CD40, and MHC class II (as shown here) molecules was determined on the surface of DC subsets. Numbers indicate percentage of cells within the gates. Blue curve (HP group, n = 6), yellow curve (P group, n = 6) and pink curve (S group, n = 6).
(3.00 MB TIF)

Table S1 Equation used to calculate endothelial permeability to albumin FITC.
(0.03 MB DOC)

Table S2 Primers for quantitative reverse-transcription polymerase chain reaction. TNF, tumor necrosis factor-α; IFN, interferon; IL, interleukin; GAPDH, glyceraldehyde-3-phosphate dehydrogenase.
(0.07 MB DOC)
