# Supplementary material for: CpG-ODN and MPLA Prevent Mortality in a Murine Model of Post-Hemorrhage-Staphyloccocus aureus Pneumonia
Source: PLoS One. 2010 Oct 7;5(10):e13228. doi: 10.1371/journal.pone.0013228 (PMC2951351; doi:10.1371/journal.pone.0013228)

Figure S1. Diagrammatic representation of the six experimental groups in the main study.

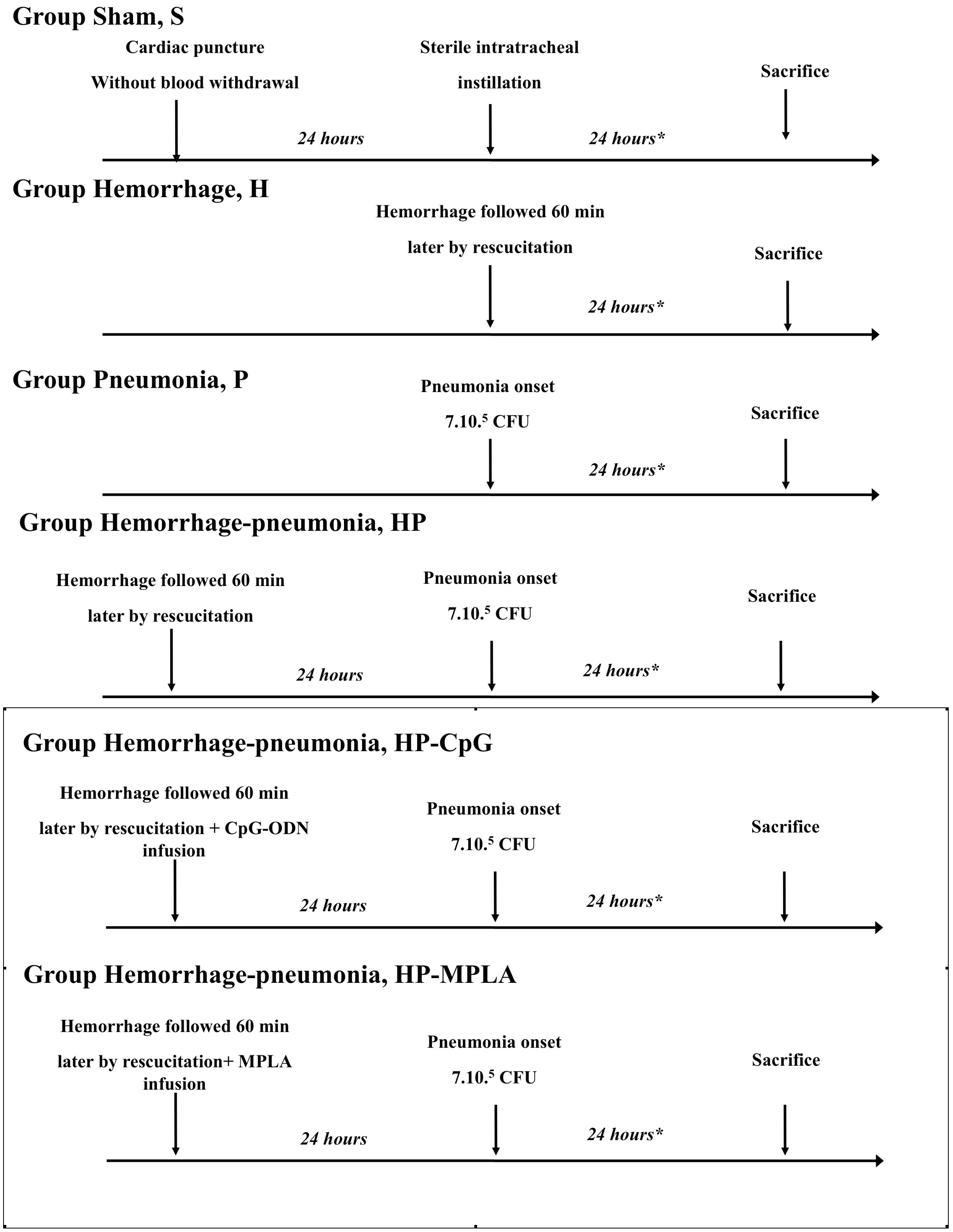

Supplement: Figure S1 — Diagrammatic representation of the six experimental groups in the main study. In the sham group (S), cardiac puncture was performed without blood collection or resuscitation. Volume-controlled hemorrhage was performed by cardiac puncture (0.3 ml/10 g body weight) and resuscitation with shed blood was performed after 60 min (groups Hemorrhage [H] and Hemorrhage-Pneumonia [HP]). After 24 hours, mice underwent intratracheal instillation of 7×10^5^ CFU (70 µl) of methicillin-susceptible S. aureus (groups Pneumonia [P] and HP) or sterile PBS (group S). Intravenous infusion of CpG-ODN (64 µg/mouse, HP-CpG group) or MPLA (50 µg/mouse, HP-MPLA group) were performed immediately after resuscitation. Twenty-four hours* after intratracheal instillation, mice were euthanized and specimens were collected. *unless otherwise stated. (1.02 MB PDF) [file pone.0013228.s001.pdf]
