# Supplementary figures and images for: CpG-ODN and MPLA Prevent Mortality in a Murine Model of Post-Hemorrhage-Staphyloccocus aureus Pneumonia
Source: PLoS One. 2010 Oct 7;5(10):e13228. doi: 10.1371/journal.pone.0013228 (PMC2951351; doi:10.1371/journal.pone.0013228)

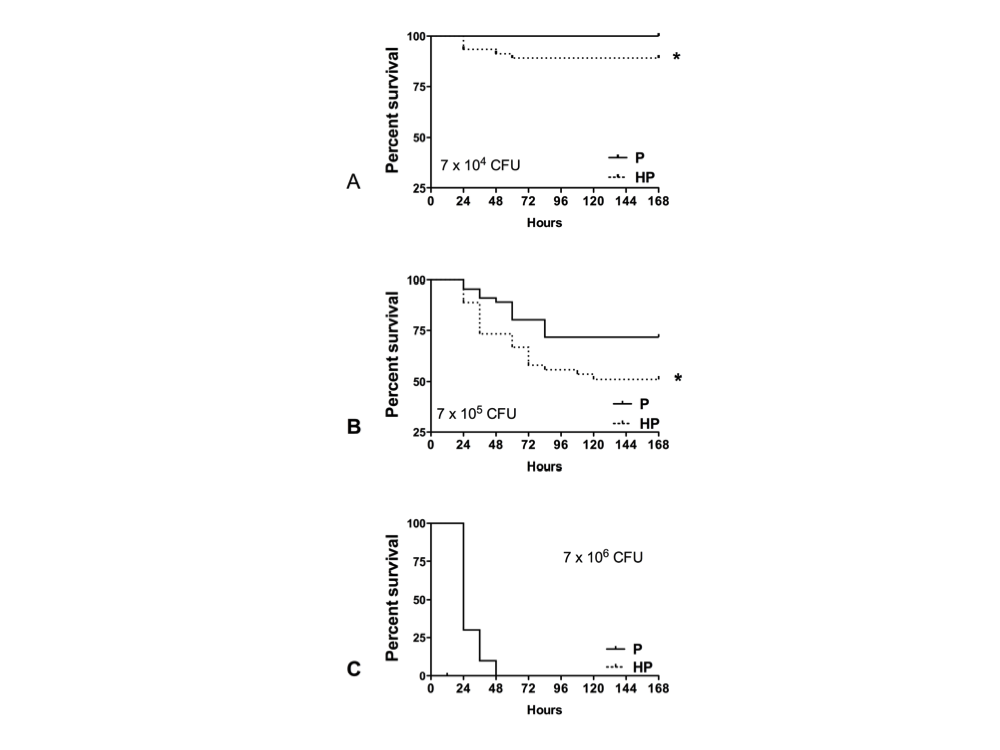

Supplement: Figure S2 — Effects of hemorrhage on inoculum-based mortality. Two groups of mice were studied: HP group (animals hemorrhaged before methicillin-susceptible S. aureus (MSSA)-induced pneumonia; n = 15) and P group (MSSA-induced pneumonia only; n = 15). Survival rates are expressed as percentage and are representative of three independent experiments. Twenty-four hours after hemorrhage for HP group, pneumonia was induced with (A) 7×10^4^ CFU, (B) 7×10^5^ CFU, or (C) 7×10^6^ CFU of MSSA. Survival was monitored twice a day for 7 days. *P<0.05 versus P group. (3.00 MB TIF) [file pone.0013228.s002.tif]

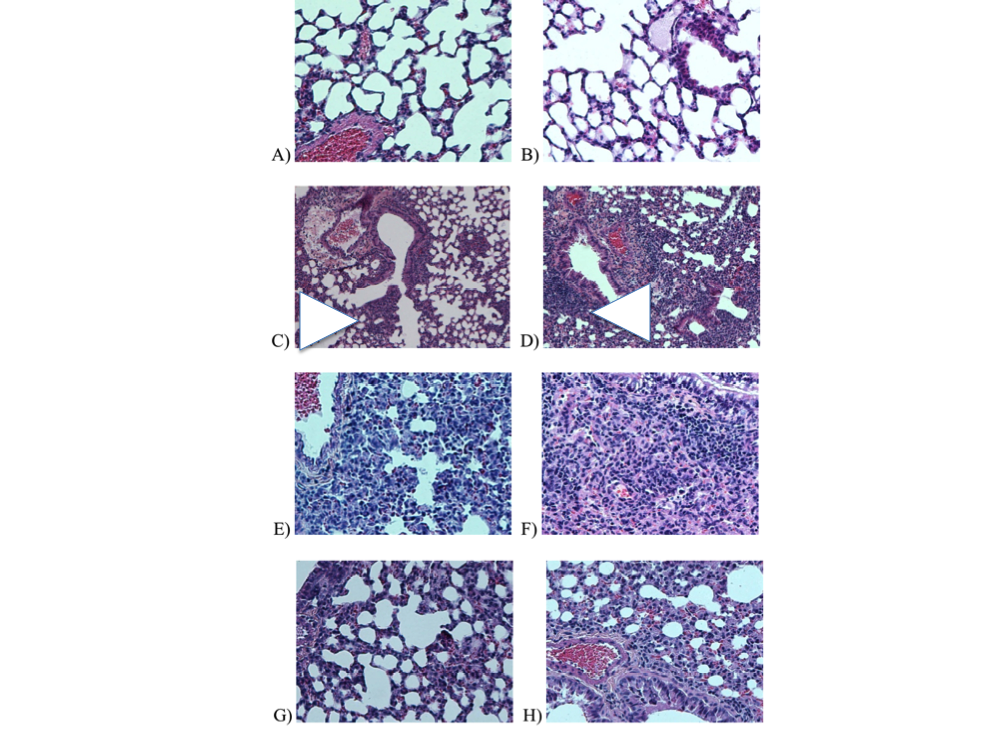

Supplement: Figure S3 — Evolution of histological findings following sepsis onset. Four groups of mice were studied (each group, n = 3): naive, sham-treated (S), methicillin-susceptible S. aureus (MSSA)-induced pneumonia only (P), and hemorrhage before MSSA-induced pneumonia (HP). Formalin-fixed tissues were processed, stained with hematoxylin and eosin, and analyzed by microscopy (magnification,×20). Representative lung histology for (A) normal lung (naive), (B) lung at 24 hours post sterile instillation (S group). The parenchyma is shown along with a series of images obtained 12, 96, and 168 hours after pneumonia onset in (C, E, G) for group P, respectively, and (D, F, H) for group HP. Aggregates of purple-stained immune cells were observed as early as 12 hours postinfection (arrow) and were more numerous in group HP compared with group P at all time points. These data established a murine model of MSSA pneumonia that closely mimics the clinical and histological findings for human patients. (3.00 MB TIF) [file pone.0013228.s003.tif]

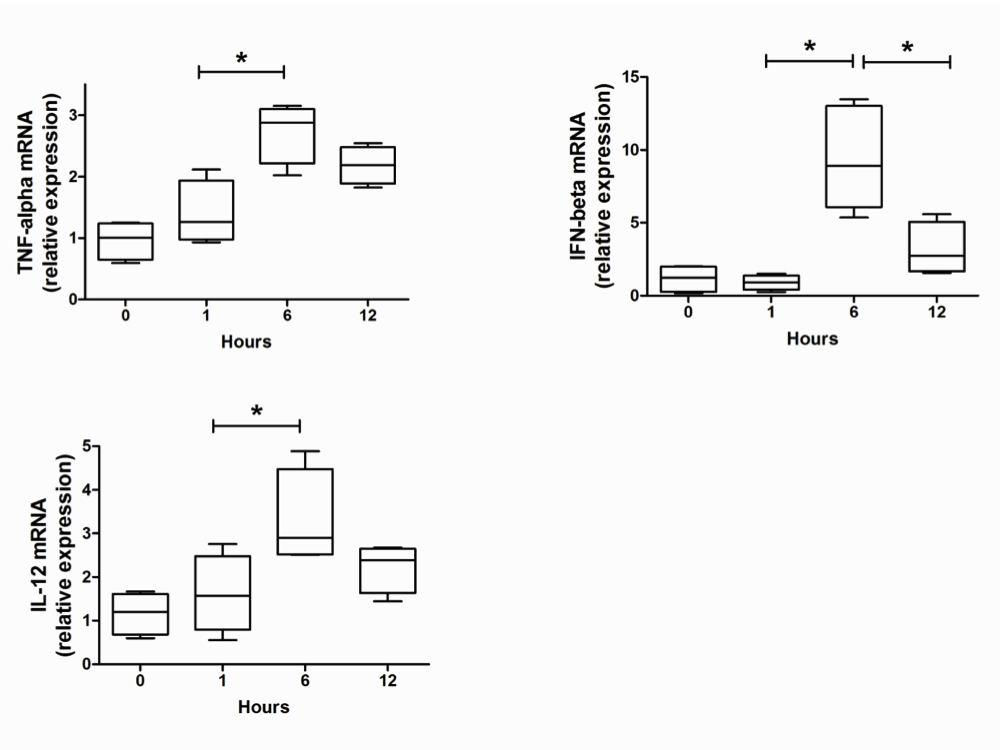

Supplement: Figure S4 — Time-dependent cytokine mRNA expression in spleen dendritic cells (DC) following sepsis onset. Mice in which pneumonia was induced by methicillin-susceptible S. aureus (P group). Mice were sacrificed 1, 6, or 12 hours after Meticillin Susceptible Staphylococcus aureus injection. Then mRNA was extracted from CD11c+ cells positively selected in spleen cells suspension. Data are representative of two independent experiments (n = 8). Boxes represent median (interquartile range). *P<0.05. (3.00 MB TIF) [file pone.0013228.s004.tif]

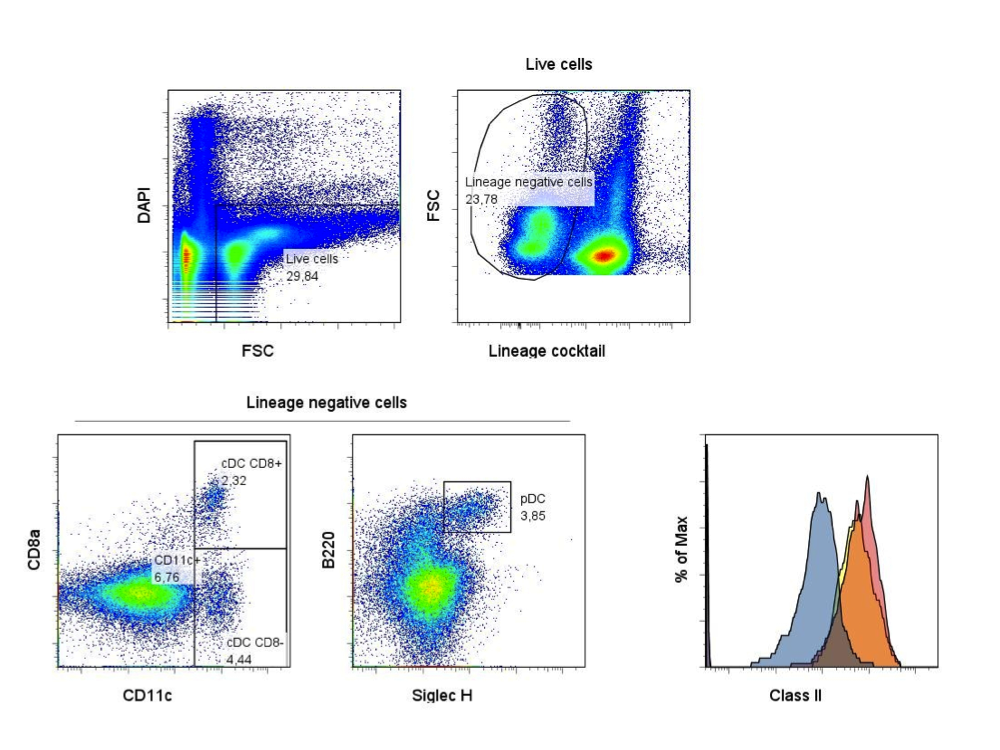

Supplement: Figure S5 — Phenotypic characterization of mouse spleen dendritic cell (DC) subsets. Spleen cells from sham-treated mice (S group), methicillin-susceptible S. aureus (MSSA)-infected mice (P group), hemorrhage-shocked and MSSA-infected mice (HP group) were labelled with antibodies against lineage antigens (CD3e, CD19, TCRb, Ter119, NK1.1) after DAPI staining. Conventional dendritic cell (cDC) and plasmacytoid cell (pDC) subsets were identified within the lineage-negative population as CD11chigh, CD8+, or CD8− cells and B220- siglec H+ cells respectively. Expression of CD80, CD86, CD40, and MHC class II (as shown here) molecules was determined on the surface of DC subsets. Numbers indicate percentage of cells within the gates. Blue curve (HP group, n = 6), yellow curve (P group, n = 6) and pink curve (S group, n = 6). (3.00 MB TIF) [file pone.0013228.s005.tif]
