# Supplementary material for: CpG-ODN and MPLA Prevent Mortality in a Murine Model of Post-Hemorrhage-Staphyloccocus aureus Pneumonia
Source: PLoS One. 2010 Oct 7;5(10):e13228. doi: 10.1371/journal.pone.0013228 (PMC2951351; doi:10.1371/journal.pone.0013228)
Supplement: Table S1 — Equation used to calculate endothelial permeability to albumin FITC. (0.03 MB DOC) [file pone.0013228.s006.doc]

**Table S1.** Equation used to calculate endothelial permeability to albumin FITC [48].

Perm-FITC (%) = **(**((FL_HS_ - FL_N_) × W_H_) – QFB**)** / **((**F_BS_ - F_BN_) × We × 0.07 × (1 – Hte)**)**

FL_HS_ is fluorescence of the lung homogenate supernatant.

FL_N_ is natural fluorescence of lung homogenate supernatant determined without administration of FITC-albumin.

F_BS_ is fluorescence of the plasma, and We × 0.07 × (1 – Hte) is the plasma volume.

F_BN_ is natural fluorescence of plasma measured without administration of FITC-albumin.

W_H_ (g) is the weight of homogenate obtained after homogenization of lungs in 1 ml of saline.

QFB is the proportion of fluorescence corresponding to intrapulmonary blood (residual intravascular blood plus that introduced into the lung interstitium via bleeding or injury).

We (g) is the mouse weight.

Hte is the hematocrit calculated from the total blood.

And QFB is calculated as described below:

1. QFB = ((FL_HS_ - FL_N_) × Hb_HS_) / Hb

Hb_HS_ (g/dl) is the residual hemoglobin measured in the homogenate supernatant.

Hb (g/dl) is the hemoglobin measured in total blood.
