# Supplementary material for: CpG-ODN and MPLA Prevent Mortality in a Murine Model of Post-Hemorrhage-Staphyloccocus aureus Pneumonia
Source: PLoS One. 2010 Oct 7;5(10):e13228. doi: 10.1371/journal.pone.0013228 (PMC2951351; doi:10.1371/journal.pone.0013228)
Supplement: Table S2 — Primers for quantitative reverse-transcription polymerase chain reaction. TNF, tumor necrosis factor-α; IFN, interferon; IL, interleukin; GAPDH, glyceraldehyde-3-phosphate dehydrogenase. (0.07 MB DOC) [file pone.0013228.s007.doc]

**Table S2.** Primers for quantitative reverse-transcription polymerase chain reaction.

| **Primer** | **Forward primer (5′-3′)** | **Reverse primer (3′-5′)** |
| --- | --- | --- |
| TNF-α | AAAGGGAGAGTGGTCAGGTTGC | GGCTGGCTCTGTGAGGAAGG |
| IFN-β | CCCTATGGAGATGACGGAGA | CTGTCTGCTGGTGGAGTTCA |
| IL-12p40 | TGTGGAATGGCGTCTCTGTCTG | CAGTTCAATGGGCAGGGTCTCC |
| IL-10 | TGGCATGAGGATCAGCAGGG | GGCAGTCCGCAGCTCTAGG |
| GAPDH | ACCACAGTCCATGCCATCAC | ACCTTGCCCACAGCCTTG |
